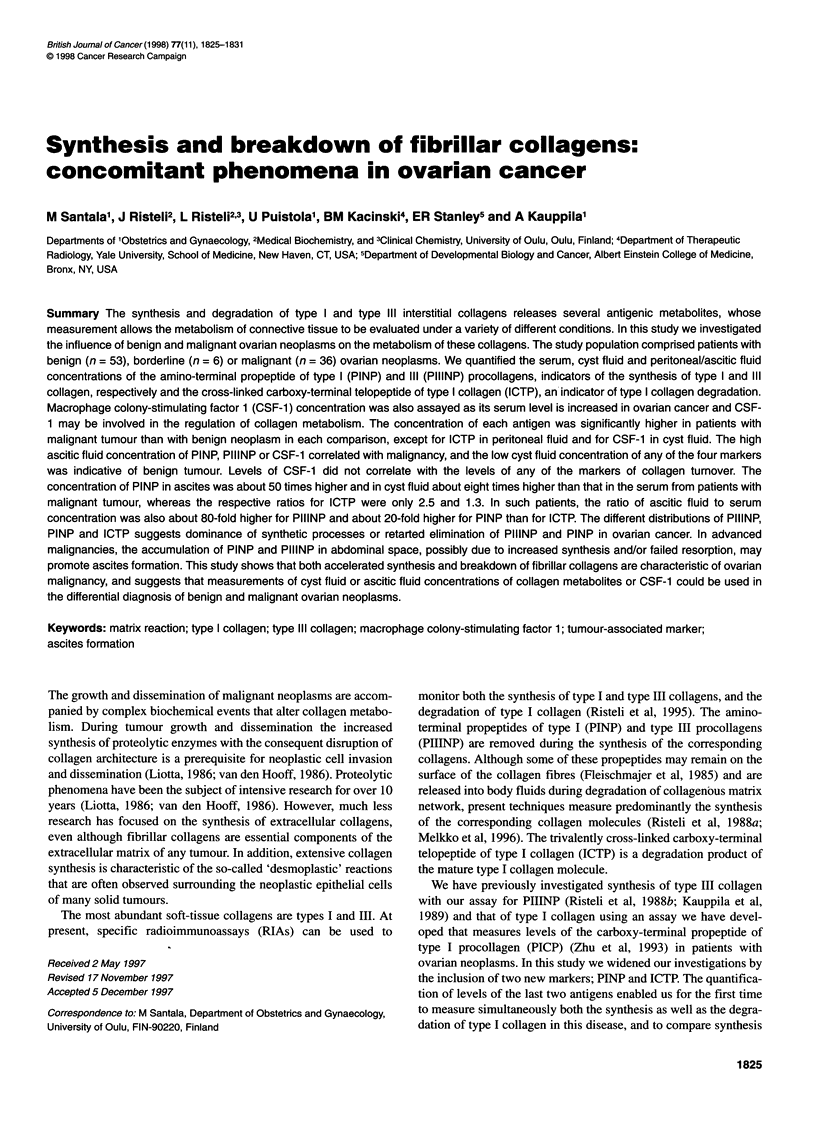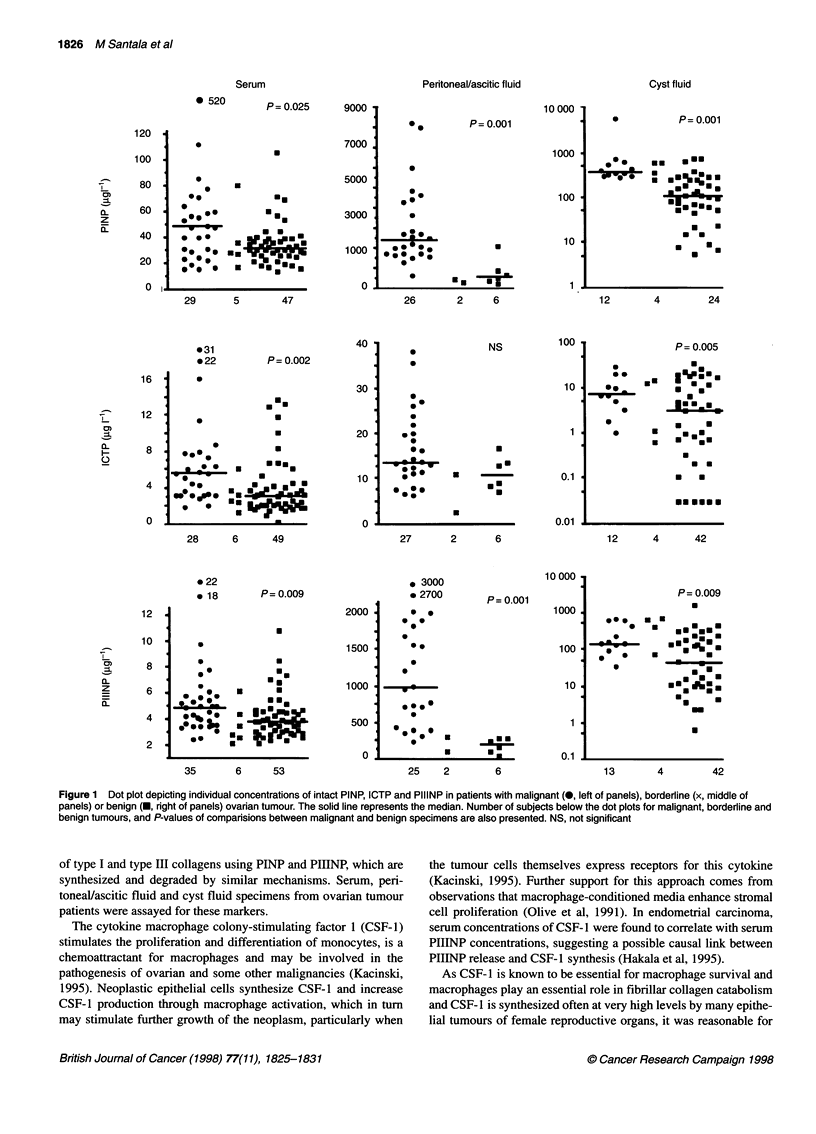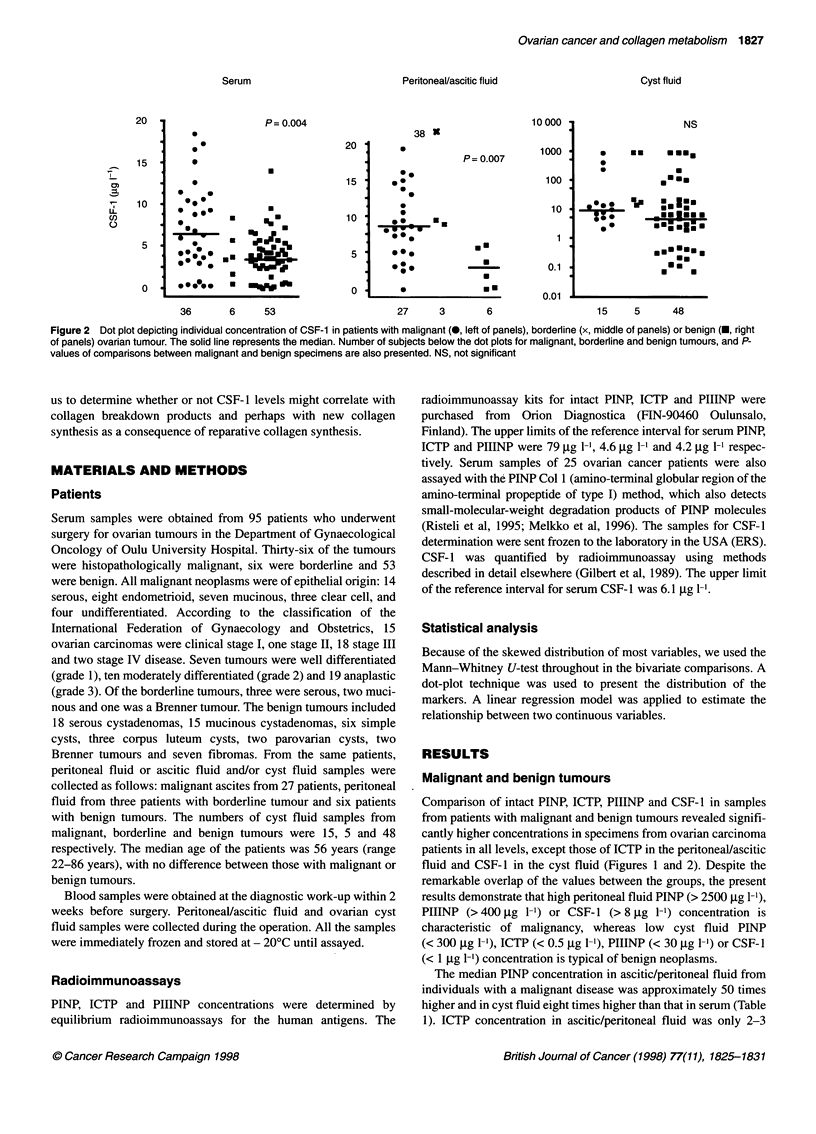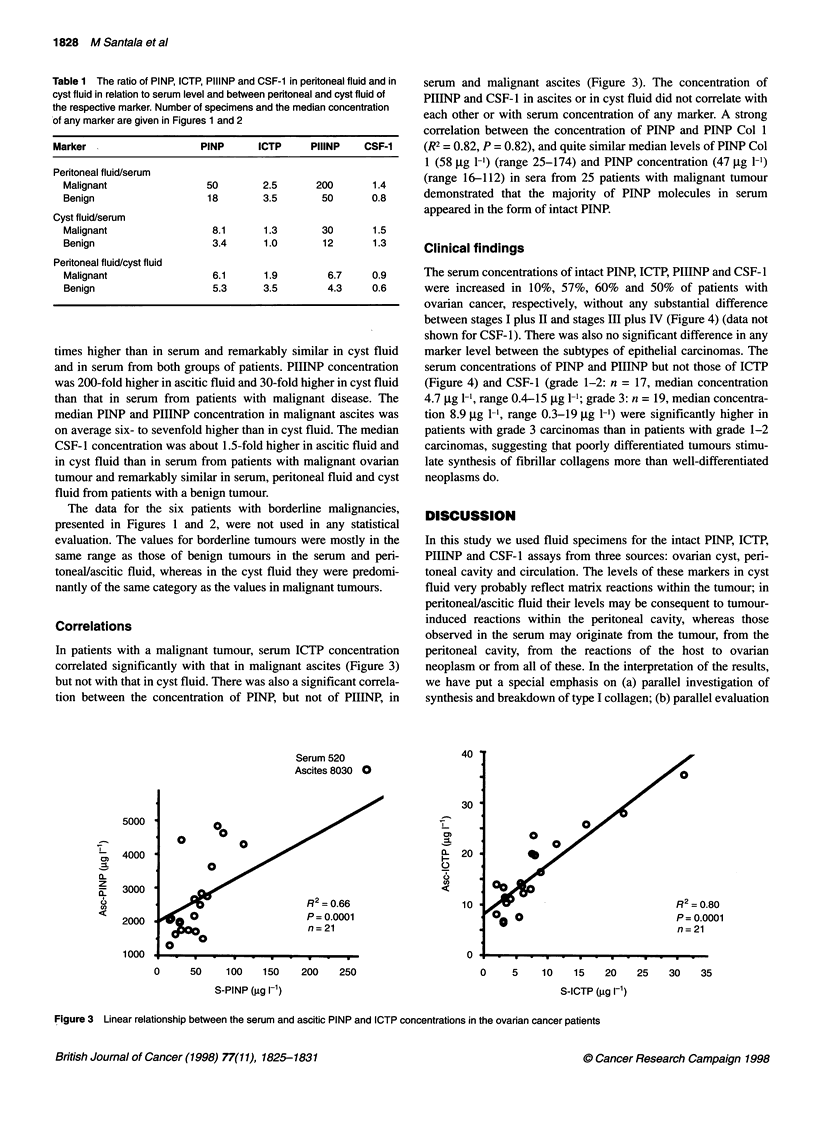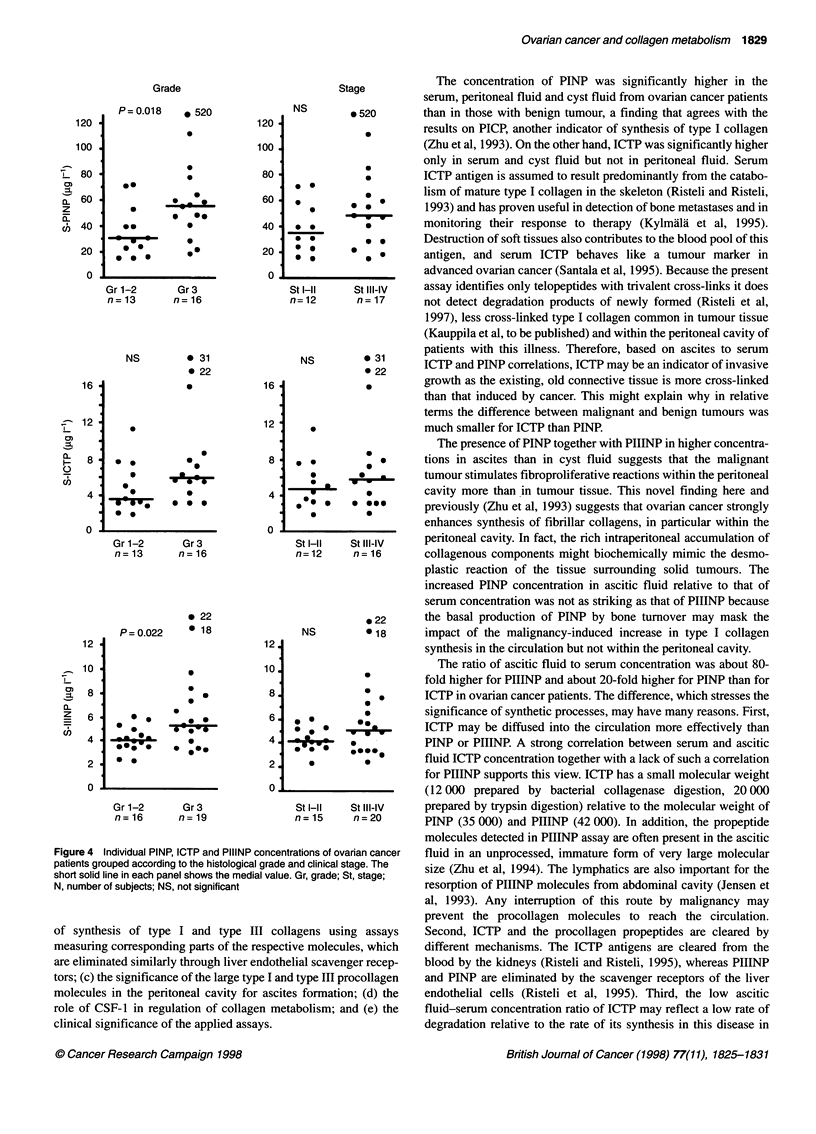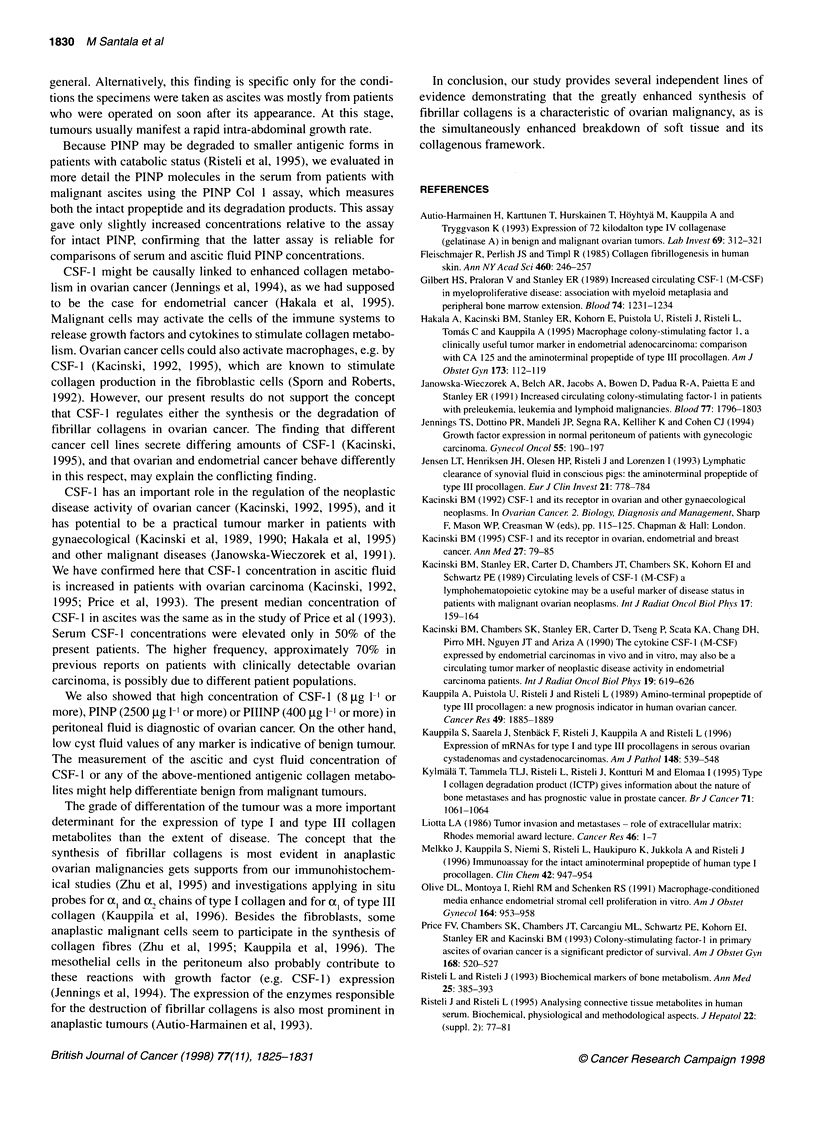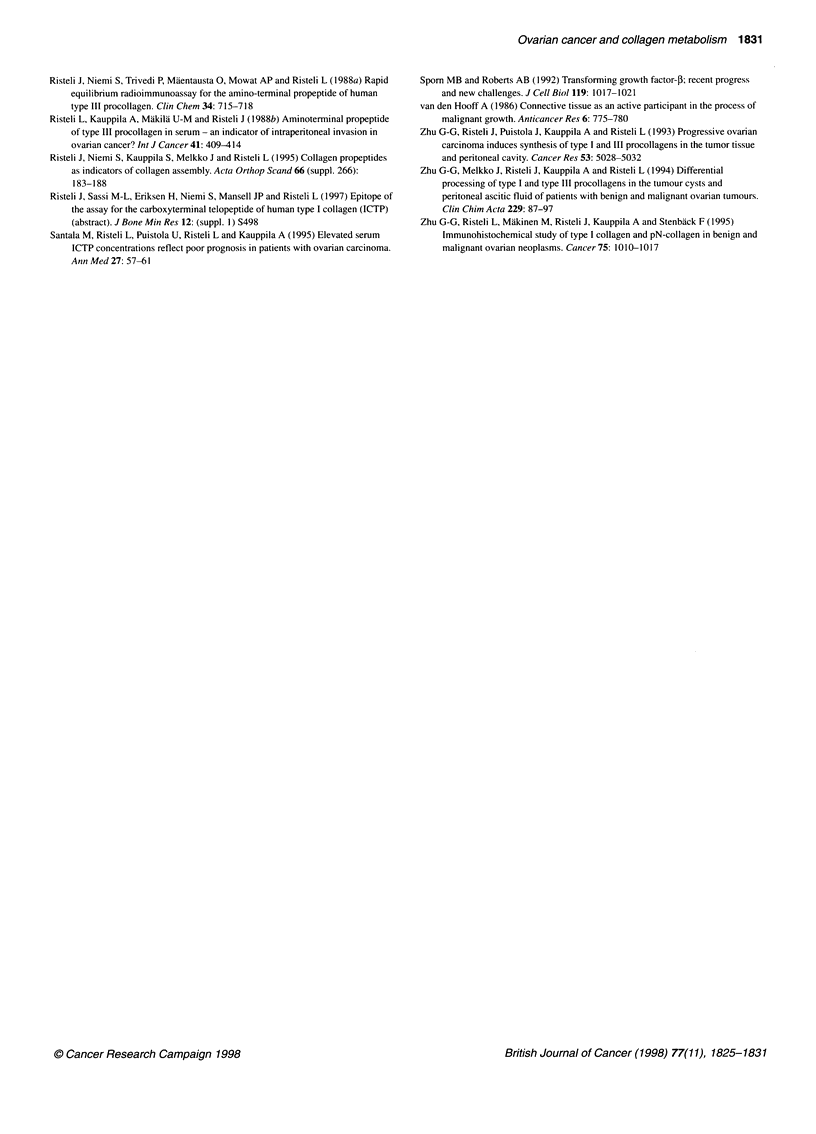# Synthesis and breakdown of fibrillar collagens: concomitant phenomena in ovarian cancer.

**DOI:** 10.1038/bjc.1998.303

**Published:** 1998-06

**Authors:** M. Santala, J. Risteli, L. Risteli, U. Puistola, B. M. Kacinski, E. R. Stanley, A. Kauppila

**Affiliations:** Department of Obstetrics and Gynaecology, University of Oulu, Finland.

## Abstract

The synthesis and degradation of type I and type III interstitial collagens releases several antigenic metabolites, whose measurement allows the metabolism of connective tissue to be evaluated under a variety of different conditions. In this study we investigated the influence of benign and malignant ovarian neoplasms on the metabolism of these collagens. The study population comprised patients with benign (n = 53), borderline (n = 6) or malignant (n = 36) ovarian neoplasms. We quantified the serum, cyst fluid and peritoneal/ascitic fluid concentrations of the amino-terminal propeptide of type I (PINP) and III (PIIINP) procollagens, indicators of the synthesis of type I and III collagen, respectively and the cross-linked carboxy-terminal telopeptide of type I collagen (ICTP), an indicator of type I collagen degradation. Macrophage colony-stimulating factor 1 (CSF-1) concentration was also assayed as its serum level is increased in ovarian cancer and CSF-1 may be involved in the regulation of collagen metabolism. The concentration of each antigen was significantly higher in patients with malignant tumour than with benign neoplasm in each comparison, except for ICTP in peritoneal fluid and for CSF-1 in cyst fluid. The high ascitic fluid concentration of PINP, PIIINP or CSF-1 correlated with malignancy, and the low cyst fluid concentration of any of the four markers was indicative of benign tumour. Levels of CSF-1 did not correlate with the levels of any of the markers of collagen turnover. The concentration of PINP in ascites was about 50 times higher and in cyst fluid about eight times higher than that in the serum from patients with malignant tumour, whereas the respective ratios for ICTP were only 2.5 and 1.3. In such patients, the ratio of ascitic fluid to serum concentration was also about 80-fold higher for PIIINP and about 20-fold higher for PINP than for ICTP. The different distributions of PIIINP, PINP and ICTP suggests dominance of synthetic processes or retarded elimination of PIIINP and PINP in ovarian cancer. In advanced malignancies, the accumulation of PINP and PIIINP in abdominal space, possibly due to increased synthesis and/or failed resorption, may promote ascites formation. This study shows that both accelerated synthesis and breakdown of fibrillar collagens are characteristic of ovarian malignancy, and suggests that measurements of cyst fluid or ascitic fluid concentrations of collagen metabolites or CSF-1 could be used in the differential diagnosis of benign and malignant ovarian neoplasms.


*British Journal of Cancer* (1998) **77**(11), 1825–1831
© 1998 Cancer Research Campaign

### Synthesis and breakdown of fibrillar collagens: concomitant phenomena in ovarian cancer

**M Santala[1], J Risteli[2], L Risteli[2,3], U Puistola[1], BM Kacinski[4], ER Stanley[5] and A Kauppila[1]**

Departments of [1]Obstetrics and Gynaecology, [2]Medical Biochemistry, and [3]Clinical Chemistry, University of Oulu, Oulu, Finland; [4]Department of Therapeutic Radiology, Yale University, School of Medicine, New Haven, CT, USA; [5]Department of Developmental Biology and Cancer, Albert Einstein College of Medicine, Bronx, NY, USA

**Summary** The synthesis and degradation of type I and type III interstitial collagens releases several antigenic metabolites, whose measurement allows the metabolism of connective tissue to be evaluated under a variety of different conditions. In this study we investigated the influence of benign and malignant ovarian neoplasms on the metabolism of these collagens. The study population comprised patients with benign ($n$ = 53), borderline ($n$ = 6) or malignant ($n$ = 36) ovarian neoplasms. We quantified the serum, cyst fluid and peritoneal/ascitic fluid concentrations of the amino-terminal propeptide of type I (PINP) and III (PIIINP) procollagens, indicators of the synthesis of type I and III collagen, respectively and the cross-linked carboxy-terminal telopeptide of type I collagen (ICTP), an indicator of type I collagen degradation. Macrophage colony-stimulating factor 1 (CSF-1) concentration was also assayed as its serum level is increased in ovarian cancer and CSF-1 may be involved in the regulation of collagen metabolism. The concentration of each antigen was significantly higher in patients with malignant tumour than with benign neoplasm in each comparison, except for ICTP in peritoneal fluid and for CSF-1 in cyst fluid. The high ascitic fluid concentration of PINP, PIIINP or CSF-1 correlated with malignancy, and the low cyst fluid concentration of any of the four markers was indicative of benign tumour. Levels of CSF-1 did not correlate with the levels of any of the markers of collagen turnover. The concentration of PINP in ascites was about 50 times higher and in cyst fluid about eight times higher than that in the serum from patients with malignant tumour, whereas the respective ratios for ICTP were only 2.5 and 1.3. In such patients, the ratio of ascitic fluid to serum concentration was also about 80-fold higher for PIIINP and about 20-fold higher for PINP than for ICTP. The different distributions of PIIINP, PINP and ICTP suggests dominance of synthetic processes or retarted elimination of PIIINP and PINP in ovarian cancer. In advanced malignancies, the accumulation of PINP and PIIINP in abdominal space, possibly due to increased synthesis and/or failed resorption, may promote ascites formation. This study shows that both accelerated synthesis and breakdown of fibrillar collagens are characteristic of ovarian malignancy, and suggests that measurements of cyst fluid or ascitic fluid concentrations of collagen metabolites or CSF-1 could be used in the differential diagnosis of benign and malignant ovarian neoplasms.

**Keywords:** matrix reaction; type I collagen; type III collagen; macrophage colony-stimulating factor 1; tumour-associated marker; ascites formation

The growth and dissemination of malignant neoplasms are accompanied by complex biochemical events that alter collagen metabolism. During tumour growth and dissemination the increased synthesis of proteolytic enzymes with the consequent disruption of collagen architecture is a prerequisite for neoplastic cell invasion and dissemination (Liotta, 1986; van den Hooff, 1986). Proteolytic phenomena have been the subject of intensive research for over 10 years (Liotta, 1986; van den Hooff, 1986). However, much less research has focused on the synthesis of extracellular collagens, even although fibrillar collagens are essential components of the extracellular matrix of any tumour. In addition, extensive collagen synthesis is characteristic of the so-called 'desmoplastic' reactions that are often observed surrounding the neoplastic epithelial cells of many solid tumours.

The most abundant soft-tissue collagens are types I and III. At present, specific radioimmunoassays (RIAs) can be used to monitor both the synthesis of type I and type III collagens, and the degradation of type I collagen (Risteli et al, 1995). The amino-terminal propeptides of type I (PINP) and type III procollagens (PIIINP) are removed during the synthesis of the corresponding collagens. Although some of these propeptides may remain on the surface of the collagen fibres (Fleischmajer et al, 1985) and are released into body fluids during degradation of collagenous matrix network, present techniques measure predominantly the synthesis of the corresponding collagen molecules (Risteli et al, 1988*a*; Melkko et al, 1996). The trivalently cross-linked carboxy-terminal telopeptide of type I collagen (ICTP) is a degradation product of the mature type I collagen molecule.

We have previously investigated synthesis of type III collagen with our assay for PIIINP (Risteli et al, 1988*b*; Kauppila et al, 1989) and that of type I collagen using an assay we have developed that measures levels of the carboxy-terminal propeptide of type I procollagen (PICP) (Zhu et al, 1993) in patients with ovarian neoplasms. In this study we widened our investigations by the inclusion of two new markers; PINP and ICTP. The quantification of levels of the last two antigens enabled us for the first time to measure simultaneously both the synthesis as well as the degradation of type I collagen in this disease, and to compare synthesis

*Received 2 May 1997*
*Revised 17 November 1997*
*Accepted 5 December 1997*

*Correspondence to:* M Santala, Department of Obstetrics and Gynaecology, University of Oulu, FIN-90220, Finland

**Figure 1** Dot plot depicting individual concentrations of intact PINP, ICTP and PIIINP in patients with malignant (●, left of panels), borderline (x, middle of panels) or benign (■, right of panels) ovarian tumour. The solid line represents the median. Number of subjects below the dot plots for malignant, borderline and benign tumours, and P-values of comparisions between malignant and benign specimens are also presented. NS, not significant of type I and type III collagens using PINP and PIIINP, which are synthesized and degraded by similar mechanisms. Serum, peritoneal/ascitic fluid and cyst fluid specimens from ovarian tumour patients were assayed for these markers.

The cytokine macrophage colony-stimulating factor 1 (CSF-1) stimulates the proliferation and differentiation of monocytes, is a chemoattractant for macrophages and may be involved in the pathogenesis of ovarian and some other malignancies (Kacinski, 1995). Neoplastic epithelial cells synthesize CSF-1 and increase CSF-1 production through macrophage activation, which in turn may stimulate further growth of the neoplasm, particularly when the tumour cells themselves express receptors for this cytokine (Kacinski, 1995). Further support for this approach comes from observations that macrophage-conditioned media enhance stromal cell proliferation (Olive et al, 1991). In endometrial carcinoma, serum concentrations of CSF-1 were found to correlate with serum PIIINP concentrations, suggesting a possible causal link between PIIINP release and CSF-1 synthesis (Hakala et al, 1995).

As CSF-1 is known to be essential for macrophage survival and macrophages play an essential role in fibrillar collagen catabolism and CSF-1 is synthesized often at very high levels by many epithelial tumours of female reproductive organs, it was reasonable for

© Cancer Research Campaign 1998

**Figure 2** Dot plot depicting individual concentration of CSF-1 in patients with malignant (●, left of panels), borderline (x, middle of panels) or benign (■, right of panels) ovarian tumour. The solid line represents the median. Number of subjects below the dot plots for malignant, borderline and benign tumours, and *P*-values of comparisons between malignant and benign specimens are also presented. NS, not significant us to determine whether or not CSF-1 levels might correlate with collagen breakdown products and perhaps with new collagen synthesis as a consequence of reparative collagen synthesis.

### MATERIALS AND METHODS

### Patients

Serum samples were obtained from 95 patients who underwent surgery for ovarian tumours in the Department of Gynaecological Oncology of Oulu University Hospital. Thirty-six of the tumours were histopathologically malignant, six were borderline and 53 were benign. All malignant neoplasms were of epithelial origin: 14 serous, eight endometrioid, seven mucinous, three clear cell, and four undifferentiated. According to the classification of the International Federation of Gynaecology and Obstetrics, 15 ovarian carcinomas were clinical stage I, one stage II, 18 stage III and two stage IV disease. Seven tumours were well differentiated (grade 1), ten moderately differentiated (grade 2) and 19 anaplastic (grade 3). Of the borderline tumours, three were serous, two mucinous and one was a Brenner tumour. The benign tumours included 18 serous cystadenomas, 15 mucinous cystadenomas, six simple cysts, three corpus luteum cysts, two parovarian cysts, two Brenner tumours and seven fibromas. From the same patients, peritoneal fluid or ascitic fluid and/or cyst fluid samples were collected as follows: malignant ascites from 27 patients, peritoneal fluid from three patients with borderline tumour and six patients with benign tumours. The numbers of cyst fluid samples from malignant, borderline and benign tumours were 15, 5 and 48 respectively. The median age of the patients was 56 years (range 22–86 years), with no difference between those with malignant or benign tumours.

Blood samples were obtained at the diagnostic work-up within 2 weeks before surgery. Peritoneal/ascitic fluid and ovarian cyst fluid samples were collected during the operation. All the samples were immediately frozen and stored at – 20°C until assayed.

### Radioimmunoassays

PINP, ICTP and PIIINP concentrations were determined by equilibrium radioimmunoassays for the human antigens. The radioimmunoassay kits for intact PINP, ICTP and PIIINP were purchased from Orion Diagnostica (FIN-90460 Oulunsalo, Finland). The upper limits of the reference interval for serum PINP, ICTP and PIIINP were 79 μg $l^{-1}$, 4.6 μg $l^{-1}$ and 4.2 μg $l^{-1}$ respectively. Serum samples of 25 ovarian cancer patients were also assayed with the PINP Col 1 (amino-terminal globular region of the amino-terminal propeptide of type I) method, which also detects small-molecular-weight degradation products of PINP molecules (Risteli et al, 1995; Melkko et al, 1996). The samples for CSF-1 determination were sent frozen to the laboratory in the USA (ERS). CSF-1 was quantified by radioimmunoassay using methods described in detail elsewhere (Gilbert et al, 1989). The upper limit of the reference interval for serum CSF-1 was 6.1 μg $l^{-1}$.

### Statistical analysis

Because of the skewed distribution of most variables, we used the Mann–Whitney *U*-test throughout in the bivariate comparisons. A dot-plot technique was used to present the distribution of the markers. A linear regression model was applied to estimate the relationship between two continuous variables.

### RESULTS

### Malignant and benign tumours

Comparison of intact PINP, ICTP, PIIINP and CSF-1 in samples from patients with malignant and benign tumours revealed significantly higher concentrations in specimens from ovarian carcinoma patients in all levels, except those of ICTP in the peritoneal/ascitic fluid and CSF-1 in the cyst fluid (Figures 1 and 2). Despite the remarkable overlap of the values between the groups, the present results demonstrate that high peritoneal fluid PINP (> 2500 μg $l^{-1}$), PIIINP (> 400 μg $l^{-1}$) or CSF-1 (> 8 μg $l^{-1}$) concentration is characteristic of malignancy, whereas low cyst fluid PINP (< 300 μg $l^{-1}$), ICTP (< 0.5 μg $l^{-1}$), PIIINP (< 30 μg $l^{-1}$) or CSF-1 (< 1 μg $l^{-1}$) concentration is typical of benign neoplasms.

The median PINP concentration in ascitic/peritoneal fluid from individuals with a malignant disease was approximately 50 times higher and in cyst fluid eight times higher than that in serum (Table 1). ICTP concentration in ascitic/peritoneal fluid was only 2–3

**Table 1** The ratio of PINP, ICTP, PIIINP and CSF-1 in peritoneal fluid and in cyst fluid in relation to serum level and between peritoneal and cyst fluid of the respective marker. Number of specimens and the median concentration of any marker are given in Figures 1 and 2

| Marker | PINP | ICTP | PIIINP | CSF-1 |
|---|---|---|---|---|
| Peritoneal fluid/serum | | | | |
| Malignant | 50 | 2.5 | 200 | 1.4 |
| Benign | 18 | 3.5 | 50 | 0.8 |
| Cyst fluid/serum | | | | |
| Malignant | 8.1 | 1.3 | 30 | 1.5 |
| Benign | 3.4 | 1.0 | 12 | 1.3 |
| Peritoneal fluid/cyst fluid | | | | |
| Malignant | 6.1 | 1.9 | 6.7 | 0.9 |
| Benign | 5.3 | 3.5 | 4.3 | 0.6 |

times higher than in serum and remarkably similar in cyst fluid and in serum from both groups of patients. PIIINP concentration was 200-fold higher in ascitic fluid and 30-fold higher in cyst fluid than that in serum from patients with malignant disease. The median PINP and PIIINP concentration in malignant ascites was on average six- to sevenfold higher than in cyst fluid. The median CSF-1 concentration was about 1.5-fold higher in ascitic fluid and in cyst fluid than in serum from patients with malignant ovarian tumour and remarkably similar in serum, peritoneal fluid and cyst fluid from patients with a benign tumour.

The data for the six patients with borderline malignancies, presented in Figures 1 and 2, were not used in any statistical evaluation. The values for borderline tumours were mostly in the same range as those of benign tumours in the serum and peritoneal/ascitic fluid, whereas in the cyst fluid they were predominantly of the same category as the values in malignant tumours.

### Correlations

In patients with a malignant tumour, serum ICTP concentration correlated significantly with that in malignant ascites (Figure 3) but not with that in cyst fluid. There was also a significant correlation between the concentration of PINP, but not of PIIINP, in serum and malignant ascites (Figure 3). The concentration of PIIINP and CSF-1 in ascites or in cyst fluid did not correlate with each other or with serum concentration of any marker. A strong correlation between the concentration of PINP and PINP Col 1 ($R^2 = 0.82$, $P = 0.82$), and quite similar median levels of PINP Col 1 (58 μg $l^{-1}$) (range 25–174) and PINP concentration (47 μg $l^{-1}$) (range 16–112) in sera from 25 patients with malignant tumour demonstrated that the majority of PINP molecules in serum appeared in the form of intact PINP.

### Clinical findings

The serum concentrations of intact PINP, ICTP, PIIINP and CSF-1 were increased in 10%, 57%, 60% and 50% of patients with ovarian cancer, respectively, without any substantial difference between stages I plus II and stages III plus IV (Figure 4) (data not shown for CSF-1). There was also no significant difference in any marker level between the subtypes of epithelial carcinomas. The serum concentrations of PINP and PIIINP but not those of ICTP (Figure 4) and CSF-1 (grade 1–2: $n = 17$, median concentration 4.7 μg $l^{-1}$, range 0.4–15 μg $l^{-1}$; grade 3: $n = 19$, median concentration 8.9 μg $l^{-1}$, range 0.3–19 μg $l^{-1}$) were significantly higher in patients with grade 3 carcinomas than in patients with grade 1–2 carcinomas, suggesting that poorly differentiated tumours stimulate synthesis of fibrillar collagens more than well-differentiated neoplasms do.

### DISCUSSION

In this study we used fluid specimens for the intact PINP, ICTP, PIIINP and CSF-1 assays from three sources: ovarian cyst, peritoneal cavity and circulation. The levels of these markers in cyst fluid very probably reflect matrix reactions within the tumour; in peritoneal/ascitic fluid their levels may be consequent to tumour-induced reactions within the peritoneal cavity, whereas those observed in the serum may originate from the tumour, from the peritoneal cavity, from the reactions of the host to ovarian neoplasm or from all of these. In the interpretation of the results, we have put a special emphasis on (a) parallel investigation of synthesis and breakdown of type I collagen; (b) parallel evaluation

**Figure 3** Linear relationship between the serum and ascitic PINP and ICTP concentrations in the ovarian cancer patients

© Cancer Research Campaign 1998

**Figure 4** Individual PINP, ICTP and PIIINP concentrations of ovarian cancer patients grouped according to the histological grade and clinical stage. The short solid line in each panel shows the medial value. Gr, grade; St, stage; N, number of subjects; NS, not significant of synthesis of type I and type III collagens using assays measuring corresponding parts of the respective molecules, which are eliminated similarly through liver endothelial scavenger receptors; (c) the significance of the large type I and type III procollagen molecules in the peritoneal cavity for ascites formation; (d) the role of CSF-1 in regulation of collagen metabolism; and (e) the clinical significance of the applied assays.

The concentration of PINP was significantly higher in the serum, peritoneal fluid and cyst fluid from ovarian cancer patients than in those with benign tumour, a finding that agrees with the results on PICP, another indicator of synthesis of type I collagen (Zhu et al, 1993). On the other hand, ICTP was significantly higher only in serum and cyst fluid but not in peritoneal fluid. Serum ICTP antigen is assumed to result predominantly from the catabolism of mature type I collagen in the skeleton (Risteli and Risteli, 1993) and has proven useful in detection of bone metastases and in monitoring their response to therapy (Kylmälä et al, 1995). Destruction of soft tissues also contributes to the blood pool of this antigen, and serum ICTP behaves like a tumour marker in advanced ovarian cancer (Santala et al, 1995). Because the present assay identifies only telopeptides with trivalent cross-links it does not detect degradation products of newly formed (Risteli et al, 1997), less cross-linked type I collagen common in tumour tissue (Kauppila et al, to be published) and within the peritoneal cavity of patients with this illness. Therefore, based on ascites to serum ICTP and PINP correlations, ICTP may be an indicator of invasive growth as the existing, old connective tissue is more cross-linked than that induced by cancer. This might explain why in relative terms the difference between malignant and benign tumours was much smaller for ICTP than PINP.

The presence of PINP together with PIIINP in higher concentrations in ascites than in cyst fluid suggests that the malignant tumour stimulates fibroproliferative reactions within the peritoneal cavity more than in tumour tissue. This novel finding here and previously (Zhu et al, 1993) suggests that ovarian cancer strongly enhances synthesis of fibrillar collagens, in particular within the peritoneal cavity. In fact, the rich intraperitoneal accumulation of collagenous components might biochemically mimic the desmoplastic reaction of the tissue surrounding solid tumours. The increased PINP concentration in ascitic fluid relative to that of serum concentration was not as striking as that of PIIINP because the basal production of PINP by bone turnover may mask the impact of the malignancy-induced increase in type I collagen synthesis in the circulation but not within the peritoneal cavity.

The ratio of ascitic fluid to serum concentration was about 80-fold higher for PIIINP and about 20-fold higher for PINP than for ICTP in ovarian cancer patients. The difference, which stresses the significance of synthetic processes, may have many reasons. First, ICTP may be diffused into the circulation more effectively than PINP or PIIINP. A strong correlation between serum and ascitic fluid ICTP concentration together with a lack of such a correlation for PIIINP supports this view. ICTP has a small molecular weight (12 000 prepared by bacterial collagenase digestion, 20 000 prepared by trypsin digestion) relative to the molecular weight of PINP (35 000) and PIIINP (42 000). In addition, the propeptide molecules detected in PIIINP assay are often present in the ascitic fluid in an unprocessed, immature form of very large molecular size (Zhu et al, 1994). The lymphatics are also important for the resorption of PIIINP molecules from abdominal cavity (Jensen et al, 1993). Any interruption of this route by malignancy may prevent the procollagen molecules to reach the circulation. Second, ICTP and the procollagen propeptides are cleared by different mechanisms. The ICTP antigens are cleared from the blood by the kidneys (Risteli and Risteli, 1995), whereas PIIINP and PINP are eliminated by the scavenger receptors of the liver endothelial cells (Risteli et al, 1995). Third, the low ascitic fluid–serum concentration ratio of ICTP may reflect a low rate of degradation relative to the rate of its synthesis in this disease in

© Cancer Research Campaign 1998

general. Alternatively, this finding is specific only for the conditions the specimens were taken as ascites was mostly from patients who were operated on soon after its appearance. At this stage, tumours usually manifest a rapid intra-abdominal growth rate.

Because PINP may be degraded to smaller antigenic forms in patients with catabolic status (Risteli et al, 1995), we evaluated in more detail the PINP molecules in the serum from patients with malignant ascites using the PINP Col 1 assay, which measures both the intact propeptide and its degradation products. This assay gave only slightly increased concentrations relative to the assay for intact PINP, confirming that the latter assay is reliable for comparisons of serum and ascitic fluid PINP concentrations.

CSF-1 might be causally linked to enhanced collagen metabolism in ovarian cancer (Jennings et al, 1994), as we had supposed to be the case for endometrial cancer (Hakala et al, 1995). Malignant cells may activate the cells of the immune systems to release growth factors and cytokines to stimulate collagen metabolism. Ovarian cancer cells could also activate macrophages, e.g. by CSF-1 (Kacinski, 1992, 1995), which are known to stimulate collagen production in the fibroblastic cells (Sporn and Roberts, 1992). However, our present results do not support the concept that CSF-1 regulates either the synthesis or the degradation of fibrillar collagens in ovarian cancer. The finding that different cancer cell lines secrete differing amounts of CSF-1 (Kacinski, 1995), and that ovarian and endometrial cancer behave differently in this respect, may explain the conflicting finding.

CSF-1 has an important role in the regulation of the neoplastic disease activity of ovarian cancer (Kacinski, 1992, 1995), and it has potential to be a practical tumour marker in patients with gynaecological (Kacinski et al, 1989, 1990; Hakala et al, 1995) and other malignant diseases (Janowska-Wieczorek et al, 1991). We have confirmed here that CSF-1 concentration in ascitic fluid is increased in patients with ovarian carcinoma (Kacinski, 1992, 1995; Price et al, 1993). The present median concentration of CSF-1 in ascites was the same as in the study of Price et al (1993). Serum CSF-1 concentrations were elevated only in 50% of the present patients. The higher frequency, approximately 70% in previous reports on patients with clinically detectable ovarian carcinoma, is possibly due to different patient populations.

We also showed that high concentration of CSF-1 (8 $\mu g\ l^{-1}$ or more), PINP (2500 $\mu g\ l^{-1}$ or more) or PIIINP (400 $\mu g\ l^{-1}$ or more) in peritoneal fluid is diagnostic of ovarian cancer. On the other hand, low cyst fluid values of any marker is indicative of benign tumour. The measurement of the ascitic and cyst fluid concentration of CSF-1 or any of the above-mentioned antigenic collagen metabolites might help differentiate benign from malignant tumours.

The grade of differentation of the tumour was a more important determinant for the expression of type I and type III collagen metabolites than the extent of disease. The concept that the synthesis of fibrillar collagens is most evident in anaplastic ovarian malignancies gets supports from our immunohistochemical studies (Zhu et al, 1995) and investigations applying in situ probes for $\alpha_1$ and $\alpha_2$ chains of type I collagen and for $\alpha_1$ of type III collagen (Kauppila et al, 1996). Besides the fibroblasts, some anaplastic malignant cells seem to participate in the synthesis of collagen fibres (Zhu et al, 1995; Kauppila et al, 1996). The mesothelial cells in the peritoneum also probably contribute to these reactions with growth factor (e.g. CSF-1) expression (Jennings et al, 1994). The expression of the enzymes responsible for the destruction of fibrillar collagens is also most prominent in anaplastic tumours (Autio-Harmainen et al, 1993).

In conclusion, our study provides several independent lines of evidence demonstrating that the greatly enhanced synthesis of fibrillar collagens is a characteristic of ovarian malignancy, as is the simultaneously enhanced breakdown of soft tissue and its collagenous framework.

### REFERENCES

Autio-Harmainen H, Karttunen T, Hurskainen T, Höyhtyä M, Kauppila A and Tryggvason K (1993) Expression of 72 kilodalton type IV collagenase (gelatinase A) in benign and malignant ovarian tumors. *Lab Invest* **69**: 312–321

Fleischmajer R, Perlish JS and Timpl R (1985) Collagen fibrillogenesis in human skin. *Ann NY Acad Sci* **460**: 246–257

Gilbert HS, Praloran V and Stanley ER (1989) Increased circulating CSF-1 (M-CSF) in myeloproliferative disease: association with myeloid metaplasia and peripheral bone marrow extension. *Blood* **74**: 1231–1234

Hakala A, Kacinski BM, Stanley ER, Kohorn E, Puistola U, Risteli J, Risteli L, Tomás C and Kauppila A (1995) Macrophage colony-stimulating factor 1, a clinically useful tumor marker in endometrial adenocarcinoma: comparison with CA 125 and the aminoterminal propeptide of type III procollagen. *Am J Obstet Gyn* **173**: 112–119

Janowska-Wieczorek A, Belch AR, Jacobs A, Bowen D, Padua R-A, Paietta E and Stanley ER (1991) Increased circulating colony-stimulating factor-1 in patients with preleukemia, leukemia and lymphoid malignancies. *Blood* **77**: 1796–1803

Jennings TS, Dottino PR, Mandeli JP, Segna RA, Kelliher K and Cohen CJ (1994) Growth factor expression in normal peritoneum of patients with gynecologic carcinoma. *Gynecol Oncol* **55**: 190–197

Jensen LT, Henriksen JH, Olesen HP, Risteli J and Lorenzen I (1993) Lymphatic clearance of synovial fluid in conscious pigs: the aminoterminal propeptide of type III procollagen. *Eur J Clin Invest* **21**: 778–784

Kacinski BM (1992) CSF-1 and its receptor in ovarian and other gynaecological neoplasms. In *Ovarian Cancer. 2. Biology, Diagnosis and Management*, Sharp F, Mason WP, Creasman W (eds), pp. 115–125. Chapman & Hall: London.

Kacinski BM (1995) CSF-1 and its receptor in ovarian, endometrial and breast cancer. *Ann Med* **27**: 79–85

Kacinski BM, Stanley ER, Carter D, Chambers JT, Chambers SK, Kohorn EI and Schwartz PE (1989) Circulating levels of CSF-1 (M-CSF) a lymphohematopoietic cytokine may be a useful marker of disease status in patients with malignant ovarian neoplasms. *Int J Radiat Oncol Biol Phys* **17**: 159–164

Kacinski BM, Chambers SK, Stanley ER, Carter D, Tseng P, Scata KA, Chang DH, Pirro MH, Nguyen JT and Ariza A (1990) The cytokine CSF-1 (M-CSF) expressed by endometrial carcinomas in vivo and in vitro, may also be a circulating tumor marker of neoplastic disease activity in endometrial carcinoma patients. *Int J Radiat Oncol Biol Phys* **19**: 619–626

Kauppila A, Puistola U, Risteli J and Risteli L (1989) Amino-terminal propeptide of type III procollagen: a new prognosis indicator in human ovarian cancer. *Cancer Res* **49**: 1885–1889

Kauppila S, Saarela J, Stenbäck F, Risteli J, Kauppila A and Risteli L (1996) Expression of mRNAs for type I and type III procollagens in serous ovarian cystadenomas and cystadenocarcinomas. *Am J Pathol* **148**: 539–548

Kylmälä T, Tammela TLJ, Risteli L, Risteli J, Kontturi M and Elomaa I (1995) Type I collagen degradation product (ICTP) gives information about the nature of bone metastases and has prognostic value in prostate cancer. *Br J Cancer* **71**: 1061–1064

Liotta LA (1986) Tumor invasion and metastases – role of extracellular matrix: Rhodes memorial award lecture. *Cancer Res* **46**: 1–7

Melkko J, Kauppila S, Niemi S, Risteli L, Haukipuro K, Jukkola A and Risteli J (1996) Immunoassay for the intact aminoterminal propeptide of human type I procollagen. *Clin Chem* **42**: 947–954

Olive DL, Montoya I, Riehl RM and Schenken RS (1991) Macrophage-conditioned media enhance endometrial stromal cell proliferation in vitro. *Am J Obstet Gynecol* **164**: 953–958

Price FV, Chambers SK, Chambers JT, Carcangiu ML, Schwartz PE, Kohorn EI, Stanley ER and Kacinski BM (1993) Colony-stimulating factor-1 in primary ascites of ovarian cancer is a significant predictor of survival. *Am J Obstet Gyn* **168**: 520–527

Risteli L and Risteli J (1993) Biochemical markers of bone metabolism. *Ann Med* **25**: 385–393

Risteli J and Risteli L (1995) Analysing connective tissue metabolites in human serum. Biochemical, physiological and methodological aspects. *J Hepatol* **22**: (suppl. 2): 77–81

© Cancer Research Campaign 1998

Risteli J, Niemi S, Trivedi P, Mäentausta O, Mowat AP and Risteli L (1988*a*) Rapid equilibrium radioimmunoassay for the amino-terminal propeptide of human type III procollagen. *Clin Chem* **34**: 715–718

Risteli L, Kauppila A, Mäkilä U-M and Risteli J (1988*b*) Aminoterminal propeptide of type III procollagen in serum – an indicator of intraperitoneal invasion in ovarian cancer? *Int J Cancer* **41**: 409–414

Risteli J, Niemi S, Kauppila S, Melkko J and Risteli L (1995) Collagen propeptides as indicators of collagen assembly. *Acta Orthop Scand* **66** (suppl. 266): 183–188

Risteli J, Sassi M-L, Eriksen H, Niemi S, Mansell JP and Risteli L (1997) Epitope of the assay for the carboxyterminal telopeptide of human type I collagen (ICTP) (abstract). *J Bone Min Res* **12**: (suppl. 1) S498

Santala M, Risteli L, Puistola U, Risteli L and Kauppila A (1995) Elevated serum ICTP concentrations reflect poor prognosis in patients with ovarian carcinoma. *Ann Med* **27**: 57–61

Sporn MB and Roberts AB (1992) Transforming growth factor-β; recent progress and new challenges. *J Cell Biol* **119**: 1017–1021

van den Hooff A (1986) Connective tissue as an active participant in the process of malignant growth. *Anticancer Res* **6**: 775–780

Zhu G-G, Risteli J, Puistola J, Kauppila A and Risteli L (1993) Progressive ovarian carcinoma induces synthesis of type I and III procollagens in the tumor tissue and peritoneal cavity. *Cancer Res* **53**: 5028–5032

Zhu G-G, Melkko J, Risteli J, Kauppila A and Risteli L (1994) Differential processing of type I and type III procollagens in the tumour cysts and peritoneal ascitic fluid of patients with benign and malignant ovarian tumours. *Clin Chim Acta* **229**: 87–97

Zhu G-G, Risteli L, Mäkinen M, Risteli J, Kauppila A and Stenbäck F (1995) Immunohistochemical study of type I collagen and pN-collagen in benign and malignant ovarian neoplasms. *Cancer* **75**: 1010–1017

© Cancer Research Campaign 1998